# Magnetic Condition-Independent 3D Joint Angle Estimation Using Inertial Sensors and Kinematic Constraints

**DOI:** 10.3390/s19245522

**Published:** 2019-12-13

**Authors:** Jung Keun Lee, Tae Hyeong Jeon

**Affiliations:** Inertial Motion Capture Lab, Department of Mechanical Engineering, Hankyong National University, Anseong 17579, Korea; yxaw12@hknu.ac.kr

**Keywords:** inertial measurement unit, joint angle, kinematic constraint, magnetic disturbance

## Abstract

In biomechanics, joint angle estimation using wearable inertial measurement units (IMUs) has been getting great popularity. However, magnetic disturbance issue is considered problematic as the disturbance can seriously degrade the accuracy of the estimated joint angles. This study proposes a magnetic condition-independent three-dimensional (3D) joint angle estimation method based on IMU signals. The proposed method is implemented in a sequential direction cosine matrix-based orientation Kalman filter (KF), which is composed of an attitude estimation KF followed by a heading estimation KF. In the heading estimation KF, an acceleration-level kinematic constraint from a spherical joint replaces the magnetometer signals for the correction procedure. Because the proposed method does not rely on the magnetometer, it is completely magnetic condition-independent and is not affected by the magnetic disturbance. For the averaged root mean squared errors of the three tests performed using a rigid two-link system, the proposed method produced 1.58°, while the conventional method with the magnetic disturbance compensation mechanism produced 5.38°, showing a higher accuracy of the proposed method in the magnetically disturbed conditions. Due to the independence of the proposed method from the magnetic condition, the proposed approach could be reliably applied in various fields that require robust 3D joint angle estimation through IMU signals in an unspecified arbitrary magnetic environment.

## 1. Introduction

Recent advances in wearable sensors and ubiquitous computing make it possible to respond to tremendous demand on health informatics related to telecare and home-monitoring for aging society. In biomechanics and rehabilitation, estimating a three-dimensional (3D) joint angle is an important requirement [1,2,3]. Marker-based optical motion capture systems have been successfully used to quantify joint kinematics by tracking the position of the surface markers during dynamic activities. However, these systems are expensive in general and suffer from occlusion. More importantly, these systems are restricted to controlled laboratory settings. It is obvious that numerous applications can tremendously benefit by continuous monitoring of joint angles in an unconstrained daily environment (e.g., outdoors) [4,5,6]. Therefore, wearable inertial sensing is an emerging technology with a growing number of potential applications in human movement analysis, as this wearable technology can overcome the in-the-lab limitation and allows the user to perform daily life activities during the measurement. 

An inertial measurement unit (IMU) typically consists of a triaxis gyroscope and a triaxis accelerometer and is often combined with a triaxis magnetometer to obtain a constant heading reference, forming an inertial and magnetic measurement unit (IMMU). Last two decades, much progress has been made in joint angle estimation using IMUs or IMMUs [1,2,5,7,8,9,10,11,12,13,14,15,16,17,18,19,20,21,22,23,24,25].

Seel et al. [7] estimated the flexion/extension angles of the knee and ankle during walking based on IMUs because the flexion/extension is the rotation around one dominant axis of the joint. El-Gohary and McNames [8] presented an unscented Kalman filter (UKF) incorporated with a kinematic arm model to track the shoulder and elbow joint angles using IMUs. Later, the UKF in [8] was improved in [5] and [9] by imposing physical constraints on the range of motion for each joint and using zero-velocity updates to mitigate the effect of the sensor drift. In [10], Vikas and Crane presented an approach to estimate the revolute and universal joint angles independent of the integration errors/drift using strategically placed accelerometers and gyroscopes. In addition, joint angle estimation using IMU signals was discussed in [13,14,15] and [20]. However, all of these studies dealt with one-dimensional or two-dimensional joint angles using IMUs, instead of 3D joint angles using IMMUs.

While magnetometers in IMMUs are required to obtain the 3D joint angles, utilization of magnetometers brings a well-known magnetic disturbance issue in angle estimation [26,27,28,29]. The magnetometer can provide a constant heading reference vector, which is the geomagnetic field vector surrounding the sensor, only when the magnetometer stays in the magnetically homogeneous environment. In recent years, researchers have developed approaches to mitigate the negative effects of the magnetic disturbance. The approaches can be placed into the two following categories: (i) the threshold-based switching approach [30,31] and (ii) disturbance model-based approach [32,33,34]. These approaches successfully presented their performances by compensating for the disturbance effects for a short time period. Nonetheless, when we deal with the joint angle estimation using wearable sensors, particularly for human motion tracking, the magnetometer can be exposed to any magnetically inhomogeneous environment. While one can stay in this environment for an extended period, none of the above methods can work for this scenario. Therefore, the magnetic disturbance is the main limitation of a magnetic-based sensing system.

This study proposes a magnetic condition-independent 3D joint angle estimation method based on IMU signals. While the proposed method estimates a 3D joint angle, it does not use a magnetometer and, instead, uses a kinematic constraint. Accordingly, it is natural that the proposed method is completely free from the magnetic disturbance issue.

The use of constraints has been proposed in previous studies [2,7,11,12,16,17,18,35,36,37,38]. Hu and Sun [35] proposed a state-constrained Kalman filter (KF) for compensation of the hard iron effect for the magnetometer based on prior knowledge, e.g., the norm of the gravity vector is 9.8 m/s^2^. Luinge et al. [16] used anatomical constraints in the elbow (i.e., abduction/adduction of the elbow is restricted to small angles) to measure the orientation of the lower arm with respect to the upper arm. They demonstrated that the constraint application could improve the accuracy of relative orientation of the upper arm and forearm. In [12], for two segments connected by a hinge joint, the heading error owing to magnetic disturbances was obtained from the projections of the joint axes into the global transverse plane. In [2,36,37], position-level constraints were used to ensure the segments remained connected at the joints. Miezal et al. [36] investigated segment orientation estimation accuracies from different methods in the presence of model calibration errors with and without using magnetometer information, for tracking a motion from a three-link chain with two spherical joints. Only minor degradation was observed when omitting the magnetometers. Furthermore, in [2], validity, test-retest reliability, and long-term stability of the 3D joint angle algorithm without magnetometer data based on the method presented in [2] were evaluated for 6 min walk tests. The results showed no systematic drift in the joint angle estimation. In [17], a velocity-level constraint of the elbow joint relative to the upper arm and forearm was imposed into the UKF to improve the estimation of the forearm and upper arm rotations. In [39], an acceleration-level constraint of a spherical joint was used to identify the joint position coordinates, i.e., the constant vectors from the joint center to the origin of the sensor frames that are attached to the segments connected by the joint. In [38], by using a similar acceleration-level kinematic constraint, a constraint-augmented KF was presented to eliminate the acceleration-induced uncertainty for a robust IMU-based attitude determination.

The approaches proposed in [11] and [18] are of our particular interest. In [18], Fasel et al. proposed a joint drift correction method for computing 3D joint angles of the knee, hip, and trunk for highly dynamic movements, e.g., in alpine skiing. The 3D accelerations measured on two adjacent segments were mapped to the connecting joint. Then, based on the two mapped acceleration vectors with respect to the global frame, the joint drift in the form of a quaternion was determined to match the two vectors. This correction method in [18] was improved in [11] by adding a second step to separately reduce the heading drift. Note that, while the constraints used in [11] and [39] are the same, the purposes of using the constraints was different, i.e., [39] to determine the constant segment-fixed vectors and [11] to estimate the drift. Because the estimated drift can be used to determine the 3D joint angle, the method in [11], [18] could be categorized into the 3D joint angle estimation. They successfully demonstrated the feasibility of the proposed methods based on the reduction of the drift caused by the strapdown integration. However, their approaches are not real-time algorithms but post-processed, which can be a critical limitation in various applications. Furthermore, while the acceleration vectors are noisy, the constraint is applied in their approaches without properly handling the noise in the vectors.

This study proposes a real-time IMU-based 3D joint angle estimation KF, where a magnetometer is replaced by a kinematic constraint. The same acceleration-level kinematic constraint in [11] derived by a spherical joint is used to provide a measurement equation for the KF. This study shows that a driftless heading estimation could be achieved in real-time by using the constraint, while the conventional approaches that rely on magnetometers are vulnerable to magnetic disturbance.

## 2. Method and Validation

### 2.1. Joint Angle Estimation Kalman Filter

The sensor signals from the accelerometer (*A*) and gyroscope (*G*) are modeled, respectively, as follows:

(1) yA=gS+aS+nA and

(2) yG=ωS+nG

where g is the gravity vector; ω is the angular velocity; a is the external acceleration; and n’s are the measurement noises, which are assumed to be uncorrelated and zero-mean white Gaussian [31]. The vectors in the sensor signals are observed in the sensor-fixed frame coordinates, as indicated by the left superscript, *S*.

In the proposed method, in order to estimate the joint angles of two links connected by a spherical joint, the 3D orientations of the two links are estimated assuming that the coordinate system of the sensor coincides with that of the link to which the sensor is attached. The coordinate system of each link is {*i*} and {*j*}, i.e., *i* and *j* ∈ *S*. An IMU having a sensor frame *S* rotates with respect to a fixed inertial reference frame, *I*. The direction cosine matrix (DCM) RSI of {*S*} with respect to {*I*} (hereafter denoted as $R_{S}$) can be interpreted as a set of three unit axis column vectors of {*I*} written in {*S*}, i.e., $X_{S}$, $Y_{S}$, and $Z_{S}$:

(3) $$R_{S}={\left[\begin{array}{ccc} X_{S} & Y_{S} & Z_{S} \end{array}\right]}^{T}.$$

When the Z-axis of the inertial frame points vertically upward (which is the most common setup), the last column $Z_{S}$ represents the attitude and can be used to calculate the roll and pitch, while the first column $X_{S}$ represents the heading and can be used to calculate the yaw, with regards to the attitude and heading reference system (AHRS). Hereafter, $Z_{S}$ and $X_{S}$ are referred to as attitude vector and heading vector, respectively. A sequential DCM-based orientation KF is considered in this study, i.e., the attitude estimation KF followed by heading estimation KF. For the attitude estimation KF for $Z_{S}$ (i.e., $Z_{i}$ and $Z_{j}$), a state-of-the-art attitude estimation algorithm having an external acceleration compensation mechanism [40] has been applied. This section discusses the heading estimation KF. For most of the orientation estimation KFs, the prediction step of the heading estimation KF for $X_{S}$ (i.e., $X_{i}$ and $X_{j}$) is based on the strapdown integration using gyroscope signals. Accordingly, the process model that updates $X_{S}$ with respect to a discrete time *t* is as follows:

(4) $$X_{S,t}=\left(I-\Delta t{\tilde{y}}_{GS,t-1}\right)X_{S,t-1}+\Delta t\left(-{\tilde{X}}_{S,t-1}\right)n_{G},$$

where **I** is the 3 × 3 identity matrix, Δt is the step size, and the overhead tilde is used to represent the standard vector cross product (i.e., $\tilde{a}=[a\times ]$ which is a 3 × 3 skew symmetric matrix) [41]. For the KF structure, (4) can be rewritten as

(5) $$x_{S,t}=\Phi_{S,t-1}x_{S,t-1}+w_{S,t-1},$$

where the state vector (lowercase) $x_{S}$ is the heading vector (uppercase) $X_{S}$. The state transition matrix $\Phi_{S,t-1}$ is $\left(I-\Delta t{\tilde{y}}_{GS,t-1}\right)$, and the white Gaussian process noise vector $w_{S,t-1}$ is $\Delta t\left(-{\tilde{X}}_{S,t-1}\right)n_{G}$. The process noise covariance matrix $Q_{t-1}$, which is defined as $E(w_{t-1}\;w_{t-1}^{T})$ (where *E* is the expectation operator), is $\Delta t\sigma_{G}^{2}{\tilde{X}}_{S,t-1}{\tilde{X}}_{S,t-1}^{T}{}_{S,t-1}$, where $\sigma_{G}^{2}$ is the gyro noise variance. 

At this stage, the a posteriori (or corrected) attitude vectors $Z_{i,t}^{+}$ and $Z_{j,t}^{+}$ from the attitude estimation KFs and the a priori (or predicted) heading vectors $X_{i,t}^{-}$ and $X_{j,t}^{-}$ from the prediction steps of the heading estimation KFs are available. Unlike most of the orientation estimation KFs, the correction step of the proposed heading estimation KF does not use magnetometer signals. It should be noted that, when the proposed method uses a kinematic constraint instead of magnetometer signals, the correction step based on the kinematic constraint is applied to only one link. In the proposed joint angle estimation method, the heading estimation KF for one link (e.g., link *i*) has only the prediction step, while that for the other link (e.g., link *j*) contains the prediction and correction steps, where the kinematic constraint is utilized. The purpose of the proposed method is to estimate the joint angle between the links and not to estimate the orientation of each link. Therefore, the following to be described is the correction step to obtain $X_{j}^{+}$ which satisfies the joint constraint, relative to the given $Z_{i,t}^{+}$, $Z_{j,t}^{+}$, and $X_{i,t}^{-}$. 

For a spherical joint allowing only relative rotation without translation, the acceleration vector at the joint center should be identical in the inertial reference frame, regardless of which IMU (attached to the preceding or following link) is used for the calculation [2]. The joint center acceleration can be thought of as the sum of the acceleration of each sensor and the acceleration owing to the rotation of that sensor around the joint center. Accordingly, the kinematic constraint can be expressed as

(6) Ri(aii+(ω˙˜ii+ω˜iiω˜ii)pii)=Rj(ajj+(ω˙˜jj+ω˜jjω˜jj)pjj),

where, for example, aii is the acceleration of the IMU attached to link *i* with respect to the sensor frame {*i*}, and pii is the constant position vector from the origin of the sensor frame {*i*} to the joint center with respect to {*i*}; thus, pii is pre-determined before the measurement. 

By applying (1) and (2) to (6), (6) becomes

(7) $$R_{i}\left(C_{i}-\varepsilon_{i}\right)=R_{j}\left(C_{j}-\varepsilon_{j}\right),$$

where the measurable vector $C_{S}$ and the error term $\varepsilon_{S}$ are as follows.

(8) CS=yAS+(y˙˜GS+y˜GSy˜GS)pSS

(9) $$\varepsilon_{S}=n_{A}+\lambda_{1,S}n_{G}+\lambda_{2,S}{\dot{n}}_{G}$$

Here, ${\dot{y}}_{GS}$ in (8) is obtained from the numerical differentiation; and λ1,S=p˜SSy˜GS−2y˜GSp˜SS and λ2,S=−p˜ss in (9). From (3), the first and second columns of $R_{S}^{T}$ are $X_{S}$ and $Y_{S}$, respectively, and $Y_{S}$ can be rewritten as ${\tilde{Z}}_{S}X_{S}$. Therefore, by transposing both sides of (7), two scalar equations can be extracted from (7) to determine unique $X_{j}^{}$ as follows.

(10) $${\left(C_{i}-\varepsilon_{i}\right)}^{T}X_{i}={\left(C_{j}-\varepsilon_{j}\right)}^{T}X_{j}$$

(11) $${\left(C_{i}-\varepsilon_{i}\right)}^{T}{\tilde{Z}}_{i}X_{i}={\left(C_{j}-\varepsilon_{j}\right)}^{T}{\tilde{Z}}_{j}X_{j}$$

In the proposed method, $X_{i}^{}$ is given as $X_{i}^{-}$ from the prediction step. In addition, by defining a posteriori attitude error vector $Z_{\varepsilon ,S}^{+}$, $Z_{S}^{}$ can be expressed as $Z_{S}^{+}-Z_{\varepsilon ,S}^{+}$. By applying these, (10) and (11) can be rewritten respectively as follows.

(12) $$C_{i}^{T}\;X_{i}^{-}-\varepsilon_{i}^{T}\;X_{i}^{-}=C_{j}^{T}\;X_{j}^{}-\varepsilon_{j}^{T}\;X_{j}^{}$$

(13) $$C_{i}^{T}\;{\tilde{Z}}_{i}^{+}X_{i}^{-}-C_{i}^{T}\;{\tilde{Z}}_{\varepsilon ,i}^{+}\;X_{i}^{-}-\varepsilon_{i}^{T}\;{\tilde{Z}}_{i}^{+}\;X_{i}^{-}=C_{j}^{T}\;{\tilde{Z}}_{j}^{+}\;X_{j}^{}-C_{j}^{T}\;{\tilde{Z}}_{\varepsilon ,j}^{+}\;X_{j}^{}-\varepsilon_{j}^{T}\;{\tilde{Z}}_{j}^{+}\;X_{j}^{}$$

For (13), the products of errors, $\varepsilon_{i}^{T}\;{\tilde{Z}}_{\varepsilon ,i}^{+}\;X_{i}^{-}$ and $\varepsilon_{j}^{T}\;{\tilde{Z}}_{\varepsilon ,j}^{+}\;X_{j}^{}$, on the left-hand-side and right-hand-side of (13), respectively, have been neglected. The error terms, $\varepsilon_{j}^{T}\;X_{j}^{}$ in (12) and $C_{j}^{T}\;{\tilde{Z}}_{\varepsilon ,j}^{+}\;X_{j}^{}$ and $\varepsilon_{j}^{T}\;{\tilde{Z}}_{j}^{+}\;X_{j}^{}$ in (13) include the unknown state $X_{j}$. By applying $X_{j}^{}=X_{j}^{-}-X_{\varepsilon ,j}^{-}$ to the error terms, where $X_{\varepsilon ,j}^{-}$ is the a priori heading error vector, (12) and (13) become

(14) $$C_{i}^{T}\;X_{i}^{-}-\varepsilon_{i}^{T}\;X_{i}^{-}=C_{j}^{T}\;X_{j}^{}-\varepsilon_{j}^{T}\;X_{j}^{-}$$

(15) $$C_{i}^{T}\;{\tilde{Z}}_{i}^{+}\;X_{i}^{-}-C_{i}^{T}\;{\tilde{Z}}_{\varepsilon ,i}^{+}\;X_{i}^{-}-\varepsilon_{i}^{T}\;{\tilde{Z}}_{i}^{+}\;X_{i}^{-}=C_{j}^{T}\;{\tilde{Z}}_{j}^{+}\;X_{j}^{}-C_{j}^{T}\;{\tilde{Z}}_{\varepsilon ,j}^{+}\;X_{j}^{-}-\varepsilon_{j}^{T}\;{\tilde{Z}}_{j}^{+}\;X_{j}^{-}$$

where the products of errors $\varepsilon_{j}^{T}\;X_{\varepsilon ,j}^{-}$ for (14) and $C_{j}^{T}\;{\tilde{Z}}_{\varepsilon ,j}^{+}\;X_{\varepsilon ,j}^{-}$ and $\varepsilon_{j}^{T}\;{\tilde{Z}}_{j}^{+}\;X_{\varepsilon ,j}^{-}$ for (15) have been neglected. 

From (14) and (15), the constraint model is obtained as follows:

(16) $$z_{t}=H_{t}x_{t}+v_{t},$$

where the state vector x, measurement vector z, observation matrix H, and white Gaussian measurement noise vector v are as follows, respectively.

(17) $$x_{t}=X_{j}$$

(18) $$z_{t}=\left[\begin{array}{c} C_{i}^{T}\;X_{i}^{-}\\ C_{i}^{T}\;{\tilde{Z}}_{i}^{+}\;X_{i}^{-} \end{array}\right]$$

(19) $$H_{t}=\left[\begin{array}{c} C_{j}^{T}\\ C_{j}^{T}\;{\tilde{Z}}_{j}^{+} \end{array}\right]$$

(20) $$v_{t}=\left[\begin{array}{c} v_{11}+v_{12}\\ v_{21}+v_{22}+v_{23}+v_{24} \end{array}\right]=\left[\begin{array}{c} \;\varepsilon_{i}^{T}\;X_{i}^{-}-\varepsilon_{j}^{T}\;X_{j}^{-}\\ C_{i}^{T}\;{\tilde{Z}}_{\varepsilon ,i}^{+}\;X_{i}^{-}+\varepsilon_{i}^{T}\;{\tilde{Z}}_{i}^{+}\;X_{i}^{-}-C_{j}^{T}\;{\tilde{Z}}_{\varepsilon ,j}^{+}\;X_{j}^{-}-\varepsilon_{j}^{T}\;{\tilde{Z}}_{j}^{+}\;X_{j}^{-} \end{array}\right]$$

It is assumed that $\varepsilon_{i}^{}$, $\varepsilon_{j}^{}$, $Z_{\varepsilon ,i}^{+}$, and $Z_{\varepsilon ,j}^{+}$ are uncorrelated each other; therefore, the measurement noise covariance matrix $M_{t}\left(=E(v_{t}\;v_{t}^{T})\right)$ is

(21) $$M_{t}=\;\;\left[\begin{array}{cc} E(v_{11}\;v_{11}^{T})+E(v_{12}\;v_{12}^{T}) & E(v_{11}\;v_{22}^{T})+E(v_{12}\;v_{24}^{T})\\ E(v_{11}\;v_{22}^{T})+E(v_{12}\;v_{24}^{T}) & E(v_{21}\;v_{21}^{T})+\ldots .+E(v_{24}\;v_{24}^{T}) \end{array}\right]$$

where

(22) $$E(v_{11}\;v_{11}^{T})={\left(X_{i}^{-}\right)}^{T}E\left(\varepsilon_{i}^{T}\;\varepsilon_{i}^{}\right)\left(X_{i}^{-}\right)$$

(23) $$E(v_{11}\;v_{22}^{T})={\left(X_{i}^{-}\right)}^{T}E\left(\varepsilon_{i}^{T}\;\varepsilon_{i}^{}\right)\left({\tilde{Z}}_{i}^{+}\;X_{i}^{-}\right)$$

(24) $$E(v_{21}\;v_{21}^{T})=E\left\{C_{i}^{T}\;{\tilde{X}}_{i}^{-}(Z_{\varepsilon ,i}^{+}){\left(Z_{\varepsilon ,i}^{+}\right)}^{T}\;{\left({\tilde{X}}_{i}^{-}\right)}^{T}\;C_{i}^{}\right\}=-C_{i}^{T}\;{\tilde{X}}_{i}^{-}\;E\left\{(Z_{\varepsilon ,i}^{+}){\left(Z_{\varepsilon ,i}^{+}\right)}^{T}\right\}\;{\tilde{X}}_{i}^{-}\;C_{i}^{}$$

For (22) and (23), $a^{T}b=b^{T}a$ is applied; and $E\left(\varepsilon_{i}^{T}\;\varepsilon_{i}^{}\right)=\sigma_{A,i}^{2}I+\sigma_{G,i}^{2}\;\lambda_{1,i}\;\lambda_{1,i}^{T}+\sigma_{Gdot,i}^{2}\;\lambda_{1,i}\;\lambda_{1,i}^{T}$, where $\sigma_{A,i}^{2}$ and $\sigma_{Gdot,i}^{2}$ are the noise variances of $y_{Ai}$ and ${\dot{y}}_{Gi}$, respectively. For (24), ${\tilde{a}}^{T}=-\tilde{a}$ is applied; and $E\left\{(Z_{\varepsilon ,i}^{+}){\left(Z_{\varepsilon ,i}^{+}\right)}^{T}\right\}$ is the covariance of the a posteriori attitude estimate, available from the attitude estimation KF. In addition, $E(v_{22}\;v_{22}^{T})$ can be obtained by replacing $X_{i}^{-}$ with ${\tilde{Z}}_{i}^{+}\;X_{i}^{-}$ in the counterpart of $E(v_{11}\;v_{11}^{T})$; and $E(v_{12}\;v_{12}^{T})$, $E(v_{12}\;v_{24}^{T})$, $E(v_{23}\;v_{23}^{T})$, and $E(v_{24}\;v_{24}^{T})$ can be obtained by replacing *i* with *j* in the counterpart of $E(v_{11}\;v_{11}^{T})$, $E(v_{11}\;v_{22}^{T})$, $E(v_{21}\;v_{21}^{T})$, and $E(v_{22}\;v_{22}^{T})$, respectively. 

**Figure 1:**
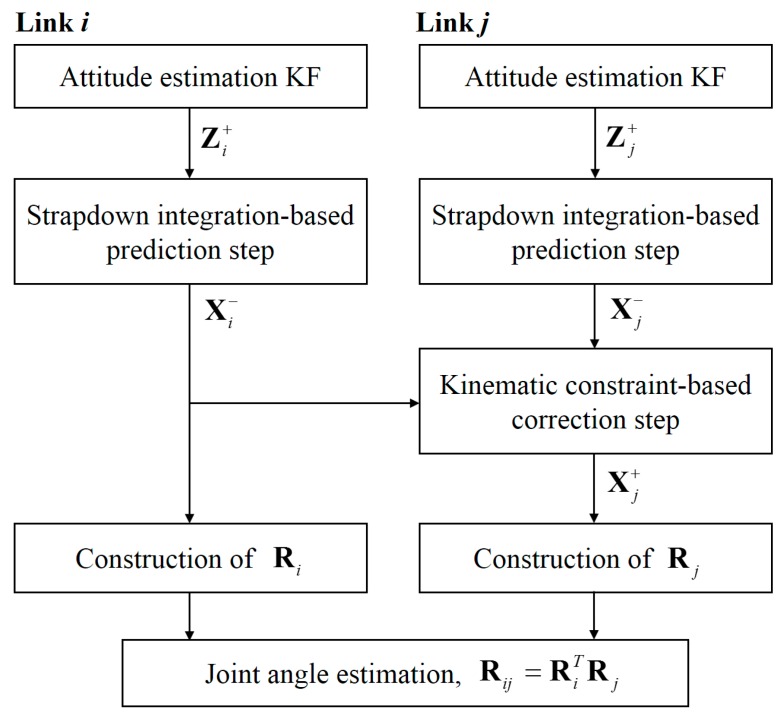
Flowchart of the proposed Kalman filter.

After the two DCMs, $R_{i}$ (from $Z_{i}^{+}$ and $X_{i}^{-}$) and $R_{j}$ (from $Z_{j}^{+}$ and $X_{j}^{+}$), are estimated, the joint angle can be extracted using the relative DCM $R_{ij}(=R_{i}^{T}\;R_{j})$ by applying the Z-Y-X Euler angle convention, i.e., $R_{ij}=R_{Z}(\alpha )\;R_{Y}(\beta )\;R_{X}(\gamma )$, where *α*, *β*, and *γ* are the yaw, pitch, and roll, respectively. The overall structure of the proposed algorithm is shown in Figure 1.

### 2.2. Validation

**Figure 2:**
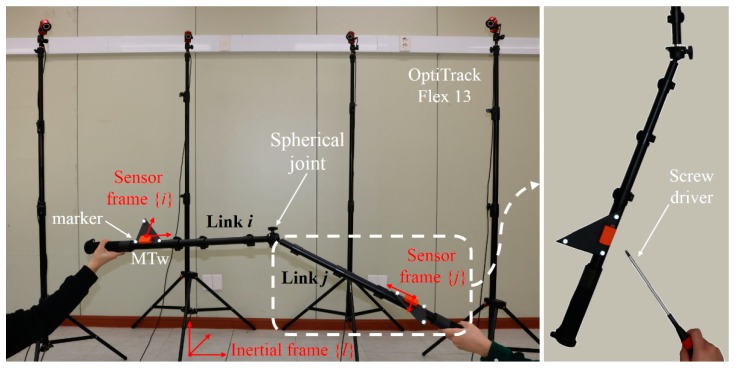
Experimental setup: (**left**) one MTw and three optical markers were attached to each link connected by a spherical joint, and (**right**) the magnetic disturbance was applied using a screwdriver.

For verification of the proposed KF, a rigid two-link system connected by a spherical joint or a ball-and-socket joint was used. Two monopods (FX-3460A from Horusbennu Inc., Korea) with a spherical joint end were utilized for the two-link system. Note that, in order to exclude any error factor related to human joints, this mechanical joint system was intentionally chosen. One MTw IMMU (from Xsens Technologies B.V., Netherlands) that includes a triaxial gyroscope, accelerometer, and magnetometer was used for each link to provide the input to the proposed algorithm operating at a 100-Hz sampling rate. To obtain the truth reference of the orientation, an OptiTrack Flex13 camera motion capture system (from NaturalPoint, Inc. USA) was used with the same sampling rate. The MTw sensor was mounted on top of a plastic right triangle ruler (with a 16.7-cm hypotenuse), and three reflective markers from the Flex13 system were attached to the vertices of the ruler using double-sided adhesive tape. These three markers formed a plane that defined a unique orientation. Then, the ruler, where the MTw and three markers were attached, was firmly fixed to each link. The constant vectors from the origin of the sensor frame to the joint center observed from the sensor frame were as follows: pii=[49.8−0.1−3.1]Tcm and pjj=[51.20.1−2.7]Tcm. These vectors were determined from the least squared method proposed in [7], see Figure 2.

Three tests were performed under kinematically dynamic and magnetically disturbed conditions, as listed in Table 1. The magnitude of the external acceleration (in m/s^2^) increased in the following order: Test 1, Test 2, and Test 3, while the magnitude of the magnetic disturbance (in arbitrary unit, a.u.) was similar for all the tests. All tests involved randomly rotating the two links manually for 180 s. The magnetic disturbance was repeatedly applied for approximately 10 s and released for approximately 30 s, to only one of the two links (i.e., link *j*) using a screwdriver with a magnetic tip.

For each of the three tests, the joint angle was estimated using four different methods. Method 1 is the conventional KF proposed by Ligorio and Sabatini [34] (estimating the attitude Z and the heading X), which is an extension of their previous work [40] (estimating Z only). Use of the same attitude estimation KF can help comparison of the performances of the different heading estimation approaches more effectively. The KF in [34] adopts a magnetic disturbance compensation mechanism based on a Gauss–Markov (GM) model for the estimation of X, in addition to an external acceleration compensation mechanism based on another GM model for the estimation of Z of the KF in [40]. Method 2 is based on the prediction of $X_{j}$ without a constraint-utilizing correction, i.e., $R_{i}$ from $Z_{i}^{+}$ and $X_{i}^{-}$ and $R_{j}$ from $Z_{j}^{+}$ and $X_{j}^{-}$. Method 2 is to see how the drift occurs when no correction is made either by the magnetometer (which is the case of Method 1) or by the kinematic constraint (which is the case of Method 3). Method 3 is the proposed method as described in the previous section. Method 4 utilizes the attitudes from the optical motion capture system (i.e., $Z_{i,opt}^{}$ and $Z_{j,opt}^{}$ that are used as the truth references), while headings $X_{i}^{}$ and $X_{j}^{}$ are estimated through the proposed method described in the previous section. Method 4 is to see the effect of the accuracy of Z on the accuracy of the joint angle. The estimation accuracy was evaluated in terms of the root mean squared error (RMSE) of the Euler angles from $R_{ij}$ (i.e., *α*, *β*, and *γ*). With regard to the noise covariance matrices, standard deviations of the accelerometer and gyroscope noises for Methods 1–3 are 14.8 mm/s^2^ and 1.5 mrad/s, respectively. Moreover, that of the magnetometer noise for Method 1 is 0.0025 a.u. and that of the derivative of the gyroscope noise for Method 3 is 25.3 mrad/s^2^.

The dimensionless model parameters used in the GM external acceleration model for Methods 1–3 were selected as *c_a1_* = 0.1 and *c_a2_* = 0.05, and those used in the GM magnetic disturbance model for Method 1 were selected as *c_m1_* = 0.1 and *c_m2_* = 0.01 (see [34] and [40] for the details of the parameters).

## 3. Results and Discussion

**Figure 3:**
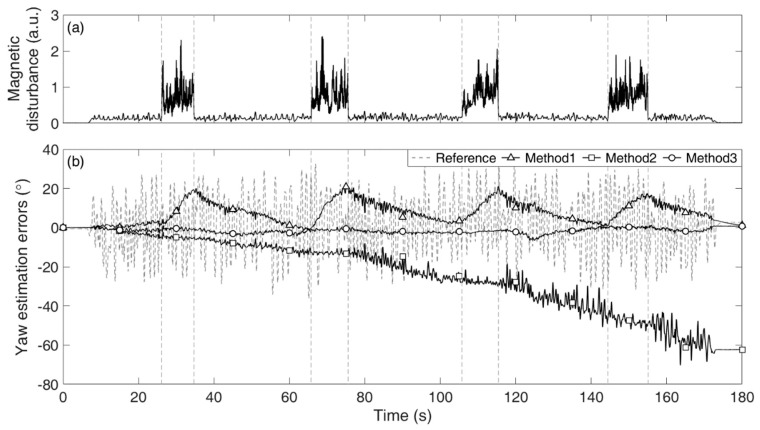
Results of the yaw estimation of the joint in Test 2: (**a**) magnitude of the applied magnetic disturbance and (**b**) estimation errors from Method 1 (*triangle*), Method 2 (*square*), and Method 3 (*circle*) with respect to the reference yaw (*dashed*).

**Figure 4:**
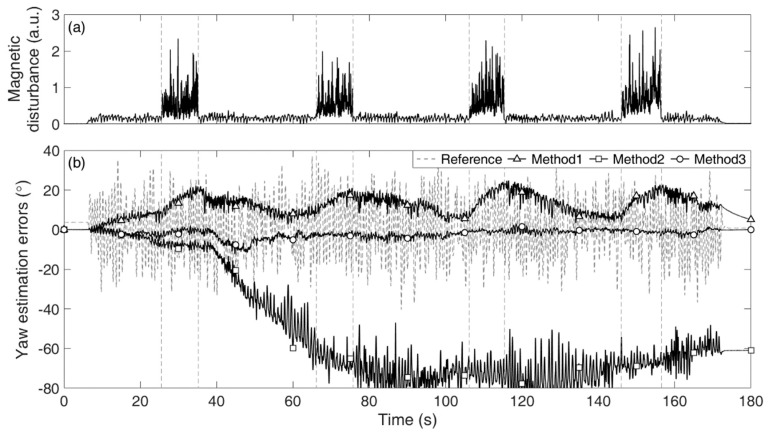
Results of the yaw estimation of the joint in Test 3: (**a**) magnitude of the applied magnetic disturbance and (**b**) estimation errors from Method 1 (*triangle*), Method 2 (*square*), and Method 3 (*circle*) with respect to the reference yaw (*dashed*).

Table 2 lists the total average and individual Euler angle RMSE values estimated from the four different methods for the three tests. Figure 3 and Figure 4 show (a) the exposed magnetic disturbance magnitudes and (b) the yaw estimation errors (*solid lines*) with respect to the reference yaw (*dashed lines*), for Tests 2 and 3, respectively.

In Test 1, the averaged RMSE from Method 1 (the conventional method) was bigger than that from Method 2 (the method where the heading estimation relied on only the prediction), while Tests 2 and 3 showed the opposite results. Because the estimation accuracy from Method 2 was dependent on the test duration and the amount of gyroscope bias, the accuracy comparison between Method 1 and Method 2 was not significantly meaningful. In all tests, Method 3 (the proposed method) outperformed Method 1 and Method 2.

As shown in Figure 3 and Figure 4, for Method 1, the estimation errors increased when the magnetic disturbances were applied, and those gradually decreased when the disturbances disappeared from the sensor. Although Method 1 had a magnetic disturbance compensation mechanism, the mechanism could not properly operate for the severely excited periods in our tests. For Method 2, the estimation errors unpredictably increased without bound, e.g., the drift error in Figure 3 developed linearly in large, while that in Figure 4 developed nonlinearly. For Method 3, the estimation errors remained small owing to the constraint. Method 3 was not affected by the magnetic disturbances at all as it did not exploit the magnetometer signals. If lower magnetic disturbance is applied, the estimation accuracy of Method 1 must be improved. It is simply natural that the estimation accuracy of Method 1 can vary considerably depending on the conditions of the magnetic disturbance. In this regard, the most important feature of the proposed method is that it is magnetic condition-independent.

**Figure 5:**
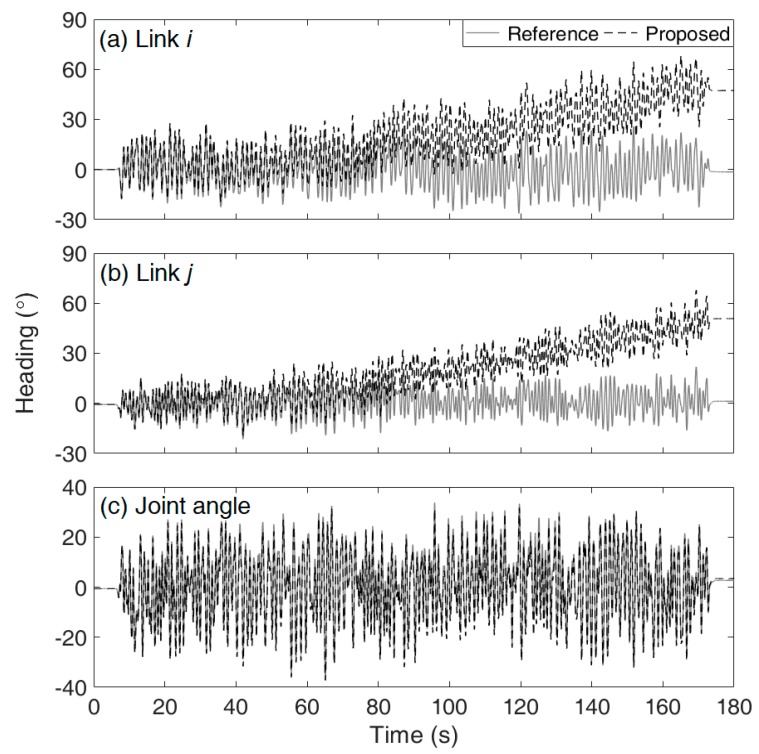
Results of heading (yaw) estimation (*dashed*) of (**a**) link *i*, (**b**) link *j*, and (**c**) the joint from Method 3 with respect to the reference heading (*solid*) in Test 2.

It should be noted that the focus of the proposed method is accurate joint angle estimation (i.e., $R_{ij}$), not the accurate estimation of individual segment orientation (i.e., $R_{i}$ or $R_{j}$). Table 3 lists the heading (yaw) estimation RMSEs of links *i* and *j* and the joint from Method 3, i.e., *α_i_* from $R_{i}$, *α_j_* from $R_{j}$, and *α_ij_* from $R_{ij}$, respectively. As an example, Figure 5 shows the result of Test 2 corresponding to the results in Table 3. As listed in Table 3, the RMSEs of *α_i_* and *α_j_* are large, while the RMSEs of *α_ij_* are small. Figure 5 shows that *α_i_* and *α_j_* drifted with time, while *α_ij_* did not. As described in Section 2.1, $R_{i}$ is determined by $Z_{i}^{+}$ and $X_{i}^{-}$, while $R_{j}$ is determined by $Z_{j}^{+}$ and $X_{j}^{+}$. Here, the heading vector of link *i*, $X_{i}^{-}$, is estimated only based on the gyroscope signals without the magnetometer signals or constraint equation and thus is drift-affected. Because the heading vector of link *j*, $X_{j}^{+}$, is estimated to satisfy the constraint at a given condition with $X_{i}^{-}$, the vector $X_{j}^{+}$ is also drift-affected. However, the relative orientation $R_{ij}$, which represents the joint angle, is free from drift. Although accurately obtaining $X_{i}^{-}$ is not required, estimating $X_{i}^{-}$ by strapdown integration using the gyroscope signals plays an important role in achieving an accurate joint angle estimation. If $X_{i}$ was fixed or arbitrarily designated based on the idea that it is not necessary to accurately obtain $X_{i}$, the results were not successful. This is because, in the heading estimation KF for $X_{j}^{+}$, the correction process based on the constraint equation has a lower burden when the predicted $X_{i}$ is applied rather than when the fixed or arbitrary $X_{i}$ is applied.

The RMSEs listed in Table 2 come from both the attitude and heading estimation errors, except for Method 4. The attitude estimation KF that was presented in [40] and used in Methods 1–3 is kinematic condition-dependent owing to the uncertainty induced by the external acceleration, despite the excellent performance of its GM-based external acceleration compensation mechanism. This could be why Test 1 had a smaller RMSE than that of Tests 2 or 3, in Method 3. For Method 4, because the reference attitude was assigned, the RMSEs represented only the heading estimation errors. When Method 3 and Method 4 were compared in terms of the averaged RMSEs, Method 4 showed a better performance than Method 3 as 0.54°, 0.47°, and 1.11° for Tests 1, 2, and 3, representing the attitude estimation errors. This result was in agreement with the acceleration magnitudes considering that Test 3 had the higher acceleration magnitude than Tests 1 and 2. The attitude estimation contained uncertainty related to the Markov chain model, while the heading estimation did not have such an uncertainty. Therefore, the measurement model derived by the acceleration-level constraint in the heading estimation was more deterministic than that based on the Gauss–Markov model in the attitude estimation, when the joint is in a dynamic condition. Even for this case, the result from Method 4 showed an error of over 1° in Test 3. The link bending resulted in a variation of the sensor-to-joint center position vector, and the noisiness of the measured joint acceleration could be one plausible explanation for this result. Note that the proposed method was validated using a mechanical two-link system, not on human bodies, on purpose. This was mainly to exclude any influence due to soft tissue artefacts and dynamic joint center variation, as a proof-of-concept.

Fasel et al. [11] stated that higher accelerations are expected to allow a more reliable estimation of joint drift because the relative impact of the small errors (e.g., originating from the sensor noise) is lower. We agree with their expectation. However, in our limited tests, this tendency was not observed. Unlike their approach, the constraint in our method was inserted into a KF platform, where the noise of the measured constraint could be optimally treated to minimize the side effects of the noise. Furthermore, in the proposed method, the constraint was inserted into the measurement system of the heading estimation KF out of the sequentially combined attitude-heading estimation KF and was not involved with the attitude estimation KF.

Although drift-free 3D joint angle estimation based on IMUs without magnetometer data was successfully achieved in [2], we believe that, unless an alternative correction process to the magnetometer-based correction is applied, the heading-related variables may be highly sensitive to the individual configuration and thus leads to unpredictable estimation, as similarly discussed in [36]. For real-time 3D joint angle estimation, this study demonstrates the replacement of a magnetometer with an acceleration-level kinematic constraint for an alternative correction process.

While this paper describes two links connected by one joint, the proposed method can be extended to finding multiple joint angles of a multi-body system. The proposed method can propagate in a sequentially recursive manner along a chain of the multi-body system. In case of a three-link system (having links *i*, *j*, and *k* where the link *i* is the floating base link), $X_{i}$ is obtained by the strapdown integration, and $X_{j}$ is determined using the kinematic constraint of the joint between the links *i* and *j*. Once $X_{j}$ is determined, $X_{k}$ is determined using the constraint of the joint between the links *j* and *k*. In this way, multiple joint angles can be accurately estimated.

A limitation of the current study could be the limited operating condition. As the proposed method uses an acceleration-level constraint of the joint, the method is only applicable when the joint is in a dynamic condition. This is also the case for the method in [11]. For static conditions, other techniques, such as zero velocity update [42,43], should be applied to prevent drift in the heading estimation.

## 4. Conclusions

This study proposes a 3D joint angle estimation method using IMUs without a magnetometer. The proposed method is implemented in a sequential DCM-based orientation KF that is composed of an attitude estimation KF followed by a heading estimation KF. In the heading estimation KF, an acceleration-level kinematic constraint from a spherical joint replaces the magnetometer signals for the correction procedure. Because the proposed method does not rely on the magnetometer, it is completely magnetic condition-independent and free from the magnetic disturbance issue.

For the averaged RMSEs for the three tests presented in this study, the proposed method produced 1.58°, while the conventional method with the magnetic disturbance compensation mechanism did 5.38°, showing a higher accuracy of the proposed method in magnetically disturbed conditions. If the attitude estimation error can be removed, the accuracy is further improved to the averaged RMSE of 0.87°, which is the case for Method 4. While the proposed method performed well in the mechanical two-link system, it needs to be validated in a real scenario, i.e., human joint angle estimation using IMUs mounted on human segments. As our future work, the proposed method will be applied to the whole body model including upper and lower limbs. Under these test setups, the influences of human joints and segments which are different from mechanical joints and links on estimation accuracy will be examined. Furthermore, computation costs of methods will be investigated.

This study demonstrated the feasibility of the proposed method, which is completely free from the magnetic disturbance issue and could provide robust joint angle estimation independent of the magnetic condition. Due to the independence of the proposed method from the magnetic condition, the proposed approach can be reliably applied in various fields that require robust 3D joint angle estimation through IMU signals in an unspecified arbitrary magnetic environment. 

## Figures and Tables

**Table 1:** Magnitudes of the external accelerations and magnetic disturbances during the tests.

	Acceleration of the Link *i* (m/s^2^)		Acceleration of the Link *j* (m/s^2^)		Disturbance of the Link *j* (a.u.)	
	Mean	Max	Mean	Max	Mean	Max
Test 1	1.28	8.22	1.30	6.11	0.23	2.60
Test 2	2.14	14.05	1.87	11.50	0.27	2.41
Test 3	5.56	37.09	4.69	18.54	0.25	2.65

**Table 2:** Root mean squared errors (RMSEs) of the 3D joint angle estimation in Tests 1–3 (unit: °).

		Roll	Pitch	Yaw	Average
Test 1	Method 1	1.52	2.23	7.87	3.87
	Method 2	0.93	0.69	1.86	1.16
	Method 3	1.16	0.56	1.10	0.94
	Method 4	0.14	0.21	0.84	0.40
Test 2	Method 1	1.48	3.81	9.16	4.82
	Method 2	6.19	9.76	31.56	15.84
	Method 3	1.53	0.86	1.99	1.46
	Method 4	0.35	0.75	1.87	0.99
Test 3	Method 1	3.51	6.06	12.77	7.45
	Method 2	14.68	19.83	58.03	30.84
	Method 3	2.65	1.49	2.86	2.33
	Method 4	0.47	0.96	2.23	1.22

**Table 3:** RMSEs of the heading estimation of each link and joint from method 3 (unit: °).

	Link *i*	Link *j*	Joint Angle
Test 1	0.59	1.53	1.10
Test 2	26.01	25.09	1.99
Test 3	65.54	64.29	2.86
